# RadArnomaly: Protecting Radar Systems from Data Manipulation Attacks

**DOI:** 10.3390/s22114259

**Published:** 2022-06-02

**Authors:** Shai Cohen, Efrat Levy, Avi Shaked, Tair Cohen, Yuval Elovici, Asaf Shabtai

**Affiliations:** 1 Department of Software and Information Systems Engineering, Ben-Gurion University of the Negev, Be’er Sheva 8410501, Israel; shco@post.bgu.ac.il (S.C.); elovici@bgu.ac.il (Y.E.); shabtaia@bgu.ac.il (A.S.); 2 Cyber Division, Elta Company, Ashdod 7710202, Israel; avishakedse@gmail.com (A.S.); tairlo@gmail.com (T.C.)

**Keywords:** radar system, anomaly detection, deep learning

## Abstract

Radar systems are mainly used for tracking aircraft, missiles, satellites, and watercraft. In many cases, information regarding the objects detected by a radar system is sent to, and used by, a peripheral consuming system, such as a missile system or a graphical user interface used by an operator. Those systems process the data stream and make real-time operational decisions based on the data received. Given this, the reliability and availability of information provided by radar systems have grown in importance. Although the field of cyber security has been continuously evolving, no prior research has focused on anomaly detection in radar systems. In this paper, we present an unsupervised deep-learning-based method for detecting anomalies in radar system data streams; we take into consideration the fact that a data stream created by a radar system is heterogeneous, i.e., it contains both numerical and categorical features with non-linear and complex relationships. We propose a novel technique that learns the correlation between numerical features and an embedding representation of categorical features in an unsupervised manner. The proposed technique, which allows for the detection of the malicious manipulation of critical fields in a data stream, is complemented by a timing-interval anomaly-detection mechanism proposed for the detection of message-dropping attempts. Real radar system data were used to evaluate the proposed method. Our experiments demonstrated the method’s high detection accuracy on a variety of data-stream manipulation attacks (an average detection rate of 88% with a false -alarm rate of 1.59%) and message-dropping attacks (an average detection rate of 92% with a false-alarm rate of 2.2%).

## 1. Introduction

Radar systems use electromagnetic radiation to detect objects within a defined scanned area [1]; they can also be used to classify the detected objects [2]. Radar systems are mainly integrated in air and terrestrial traffic-control systems [3], autonomous vehicles [4], air-defense systems, anti-missile systems, aircraft anti-collision systems, and ocean surveillance systems [5].

In recent years, as technology has evolved, the use of radar systems has increased along with a reliance on their correct and reliable operation. Unfortunately, radar systems are vulnerable to cyber attacks [6].

Radar systems often include extended sets of components, such as communication systems and SCADA systems. These components can be exploited by attackers in order to compromise a radar system [7]. In addition, in many cases, radar systems are integrated within systems that are vulnerable to cyber attacks, such as autonomous vessels [8] and smart vehicles [9]. These vulnerabilities may be used by an attacker as a back door for an attack on a radar system.

Typically, the radar system architecture consists of the following basic components: (1) an antenna responsible for transmitting/receiving electromagnetic waves to/from a scanned area and (2) a radar controller responsible for analyzing the waves received from the antenna in order to determine the properties of an object.

Usually, a radar controller is connected to a centralized switch that controls the routing of a radar system’s messages. In many cases, the switch can be used to communicate with external entities, i.e., other systems. Often, information regarding the objects detected by a radar system is sent to, and used by, a peripheral consuming system, such as a missile system or a graphical user interface used by an operator [10]. Those systems process the data stream and make real-time operational decisions based upon the data received. Detecting anomalies in a radar data stream ensures the potential catching of manipulations conducted at different stages of signal acquisition and processing. This may be in addition to potentially catching manipulation threats that are commonly raised during data-stream transmissions to peripheral consuming systems. To the best of our knowledge, no study has proposed a method for detecting anomalies in radar-system data streams.

In this study, we address this gap. We propose an unsupervised deep-learning-based method for detecting anomalies in critical parts of the data stream. In contrast to rule-based detection methods, our method does not require any prior knowledge of the malicious-manipulation model; it considers general attack scenarios in which an attacker manipulates the data that are being generated by a radar system and transferred to peripheral consuming systems. Because such manipulation can occur when data are in motion or in use, we also consider adversaries that can bypass cryptography-based defenses if such defenses exist, e.g., in cases in which higher levels of software are compromised. The proposed method simply requires the ability to monitor messages sent by a radar controller, and thus, it can be easily and safely integrated into existing systems without the need to change the systems, e.g., software, hardware, and internal communication protocols. Our proposed method was designed to deal with the particular data comprising the radar data stream; unlike many other domains, radar data streams are made up of sequential and heterogeneous data.

In order to evaluate the proposed method, we used data collected from four real radar systems. We collected legitimate data streams into which we injected a variety of artificial attacks, including manipulations with high impacts on a system (integrity violations and denials of service), to assess our method’s performance. The results of our evaluation showed that our proposed method can detect 90% of message-dropping attacks with a false-positive rate of 2% and can detect from 76% to 96% of feature-manipulation attacks with a false-positive rate of 2%. These results were obtained in a cross-session experiment in which the model was trained on data collected from three radar systems and tested on data collected from another radar system, thus demonstrating the ability to migrate a pretrained model to new radar systems without retraining.

We summarize the main contributions of this study as follows:

- We present a deep-learning-based method that consists of two modules to detect anomalies in critical parts of the heterogeneous data stream generated by radar systems.
- The proposed method can be integrated into existing radar systems without changing the radar systems’ existing components and without retraining the model.
- The structure of the proposed method enables it to identify an anomaly type detected, determine whether a legitimate message is missing from a sequence of messages, and understand whether a feature of an existing message has been manipulated.
- The effectiveness of the proposed method was demonstrated in experiments using a dataset collected from real operational radar systems and simulated attacks.

## 2. Background on Radar Systems

A radar system uses radio waves to determine both the location of an object relative to a system and the distance between the object and the system. It operates by transmitting electromagnetic signals in a certain direction and monitoring the signals that are reflected back from objects. The reflected signals are then sent to a signal processor, which analyzes the signals and aims to extract the properties of the objects [10].

### 2.1. System Components

**Figure 1:**
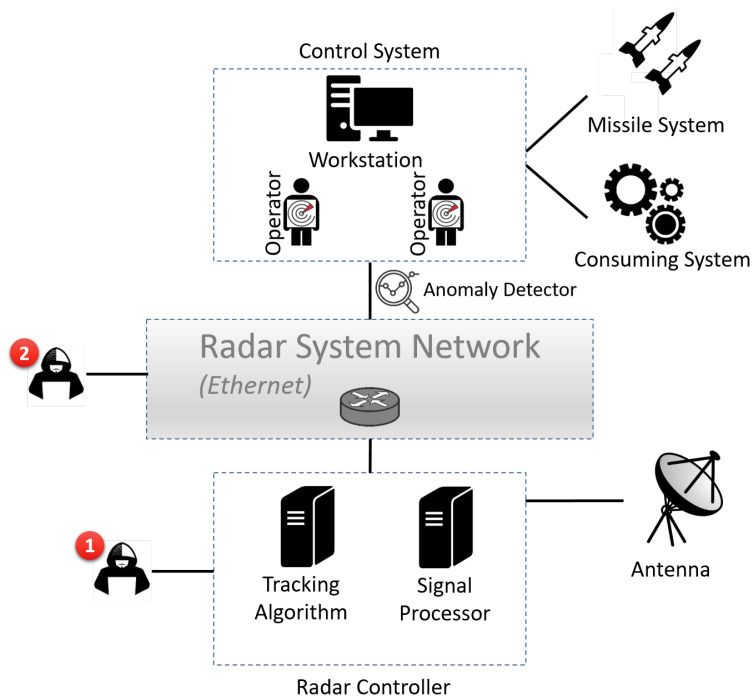
An example of a typical radar-system architecture. The red circles indicate our assumed adversary’s locations. Our proposed anomaly-detection method is located at the link between the radar system and the consuming systems.

A typical radar system architecture includes the following components (presented in Figure 1):

- **Antenna:** An antenna transforms an electric current into electromagnetic waves and vice versa (conducts transmission and reception, respectively). Usually, one bidirectional antenna is used; however, radar systems with two separate antennas (a transmitting antenna and a receiving antenna) also exist.
- **Radar controller:** A radar controller consists of two main components: (1) a signal processor, which receives signals from an antenna (usually via an optical link) and analyzes the signals using a signal-processing algorithm aimed at identifying potential relevant objects, and (2) a tracking algorithm, which analyzes a signal-processor’s output with the aim of classifying objects and tracking movements.
- **Control system:** A control system is responsible for analyzing radar yields and activating the different systems connected to a radar system; for example, it may activate a weapon system to neutralize a detected threat.
- **Radar-system network:** A radar-system network is used as a communication channel between a radar controller and a control system.

### 2.2. Data-Stream Description

A radar system (radar controller) continuously sends a stream of messages to peripheral consuming systems. The consuming systems process the data stream and make real-time operational decisions based on the data received. Each message contains a plot record. This record describes a detected object at a given time.

**Figure 2:**
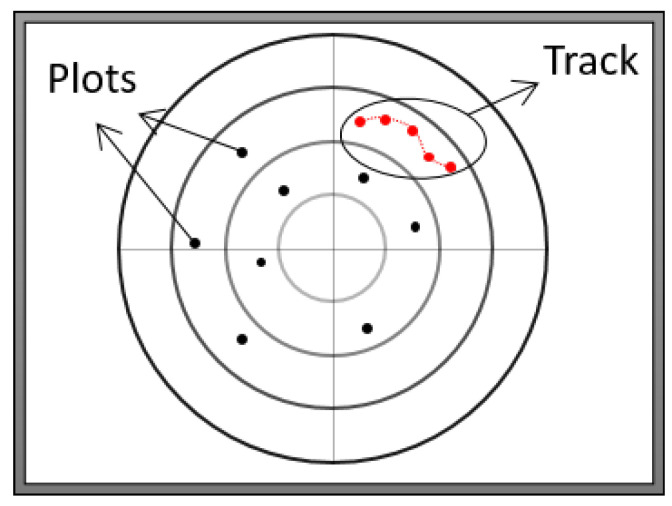
Objects identified at a specific timestamp are represented by points. A sequence of points related to the same detected object are represented by a path of interconnected points, i.e., a track.

Since the radar system continuously scans the defined area, several plots may relate to the same object, describing its properties at different points in time. A sequence of plots related to the same object is defined as a track. Each plot may be correlated with other plots, e.g., when they are part of an identified track. Each track is associated with an identification number (referred to as a track ID). Thus, the track ID is a part of each individual plot record. Figure 2 illustrates a set of plots (individual points) and a track (sequence of connected plots). Each plot record contains the following attributes:

- **Metadata:** Contains the source ID (unique identifier of a sending radar system), message ID, message length, etc.
- **Identified object’s properties:** Categorical and numerical attributes that describe an identified object, such as the detected object’s location, speed, or type, e.g., airplane or bird.
- **Sequence-related properties:** Describe different properties of the plot record as part of a sequence of plots related to the same object. One property is the identification number of the track that a plot belongs to. Another property is a timestamp indicating when a plot has been updated on a system; this property describes the delay between a previous plot and a current plot.

## 3. Threat Model

Radar systems are vulnerable to a variety of threats [6]. In this study, we considered attack scenarios in which an attacker manipulates the data that is being generated by a radar system and transferred to peripheral consuming systems. Such a manipulation may occur when data are in motion or in use. Therefore, we considered adversaries that can bypass cryptography-based defenses if any such defenses are present. Specifically, as illustrated in Figure 1, the adversary locations (points of manipulation) considered in this study were a radar controller and a radar-system network. They can be compromised with physical or remote access or through a supply-chain attack, i.e., before the radar system’s deployment.

Once a radar controller has been compromised by an attacker (whether remotely or through a supply-chain attack), the attacker can execute malicious commands that manipulate data while they are being processed—prior to their transmission from the radar controller. With physical access to a radar-system network, an attacker can replace a networking device, e.g., switch, with a malicious networking device that manipulates data in motion. A similar threat exists when a radar-system network is compromised by remote attackers. The effects of such tampering attempts are expected to be covert and, more specifically, to be “under the radar” of radar operators, i.e., radar operators should not be able to identify the tampering.

Based on the specified threat model, we suggest an anomaly detection method that (1) detects abnormal data behaviors indicative of such malicious activity and (2) can be implemented as a detector at the link between a radar-system network and a control system to provide anomaly-related insights to radar operators and/or systems that consume radar data.

## 4. Related Work

Most studies that used machine-learning techniques to protect radar systems from attacks focused on protecting the systems from jamming attacks since they are the main threats to radar systems. Some studies proposed jamming classification methods [11,12,13,14], and others proposed jamming mitigation strategies [15,16,17]. In addition, a comprehensive survey discussing all of these methods in detail was published [18]. In other related research, machine-learning techniques were used to protect the systems generally integrated within radar systems, such as the ADS-B [19,20] and AIS systems [21].

To the best of our knowledge, no study has proposed a method for detecting anomalies in radar-system data streams. Detecting anomalies in a radar data stream can allow one to potentially catch manipulations conducted at different stages of signal acquisition and processing. This may occur in addition to potentially catching a manipulation threat that is naturally raised during transferring data to peripheral consuming systems.

One big advantage of neural networks is their ability to learn non-linear and complex relationships between input features in an unsupervised manner. Not surprisingly, in recent years, deep-learning models have been used increasingly to detect anomalies in the cyber domain. Most of the methods proposed are based on autoencoders and their variants [22]. An autoencoder (AE) is an unsupervised algorithm that compresses an input into a lower dimensionality and then decompresses the input into its original dimensionality; thus, normal instances are decompressed properly, while abnormal instances are not. In this way, anomalous inputs can be identified. Some examples of methods based on AEs include N-BIoT [23], which uses an AE to detect botnet behaviors in the network traffic of IoT devices, and Kitsune [24], which utilizes a smart feature mapper and an ensemble of AEs to detect anomalous behaviors in network traffic.

LSTM-based networks are also widely used to detect anomalies in network traffic; these deep-learning networks are very well-suited for sequential data. In this type of network, instead of processing a whole sequence together, a network processes each object in a sequence separately in chronological order. Thus, in each step, the network’s inputs are (1) the current object in the sequence along with (2) the output of the network from the previous step. An example of using an LSTM-based network was presented by Bontemps et al. [25], who predicted anomalies in the network data stream by using a sample as well as the context of the sample.

Previous studies that used unsupervised deep-learning models to detect anomalies in network traffic relied strictly on numerical features. However, in this study, we took into consideration the fact that a data stream created by a radar system is heterogeneous, i.e., it contains both numerical and categorical features. Such features, which are used to identify the different properties of a physical object, have non-linear and complex relationships. Since manipulating both types of features may significantly violate the integrity and availability of radar systems, there is a need for a deep-learning method designed to detect such activities in real time.

In contrast to rule-based detection methods, our method does not require any prior knowledge of the malicious manipulation model; our proposed method is based on an unsupervised learning technique and is designed to efficiently detect attack scenarios targeting the integrity and availability of radar systems. Moreover, we showed that trained models can be transferred from one radar system to another radar system without retraining.

Model-based and machine-learning-based anomaly detection methods were also proposed for common industrial control systems [26,27]. Liu et al. [26] presented a detection strategy that uses one detector to deal with integrity and availability attacks for the tracking of industrial-control cyber-physical systems (ICPS); their method, which is based on quantifying the dynamic variations of a generalized model implied by operating data, can be deployed independently in an active ICPS and does not cause any loss of control performance. Houng et al. [27] proposed a federated learning approach for detecting anomalies in time series data for industrial-control systems.

## 5. High-Level Description of the Proposed Method

We propose an unsupervised anomaly detection method that is based on the continuous monitoring of messages transmitted from radar to peripheral consuming systems. Our proposed method utilizes state-of-the-art deep-learning modules to detect possible malicious data manipulation. In this section, we provide a high-level description of each proposed module: the main role, motivation, and general operation.

### 5.1. Description of the Proposed Method

**Figure 3:**
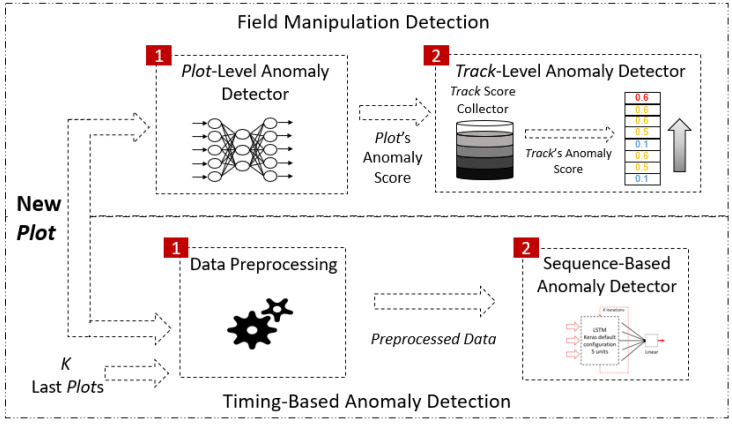
High-level architecture of the proposed anomaly detection method. Our method consists of two modules: a field manipulation-detection module and a timing-based anomaly-detection module.

The proposed anomaly detection method (see Figure 3) consists of two main modules: (1) a field manipulation-detection module and (2) a timing-based anomaly-detection module. Each module outputs an anomaly score, and an alert is generated if the score exceeds a predefined threshold.

- **Field manipulation detection:** For each track, the field manipulation-detection module receives the categorical and numerical features of a plot. First, it determines if the relationships between the features’ values are anomalous. If so, an anomaly score for the plot is generated. An anomalous relationship between the values of a plot’s features indicates that a malicious change has been made aimed at violating the radar system’s integrity. Such manipulations may cause consuming systems or radar operators to incorrectly characterize and handle detected objects. Finally, to increase the sensitivity of the detection method for malicious manipulation attacks, an alert is generated if the score’s average exceeds a certain threshold. The latter step aims to detect anomalies that are harder to detect by analyzing a single plot.
- **Timing-based anomaly detection:** The timing-based anomaly detection module receives the last *K*-inspected plots and the current plot associated with the same track and determines if the time difference between the current plot and the previous plot is anomalous. An anomalous time difference between plots indicates a malicious plot dropping or injecting of events. Such activities may enable an object to evade detection by the detection system or cause a denial of service.

### 5.2. Design Considerations

The following considerations were taken into account in the design of the proposed method:

1. **Analysis of heterogeneous data:** As mentioned in Section 2.2, plot records consist of numerical and categorical features. They are used by a radar system to identify an object’s properties.One big advantage of deep-learning models is their ability to learn and model non-linear and complex relationships between input features. When categorical features are properly encoded, such relationships can be learned when the given data are heterogeneous.
2. **Immediate detection:** Often, radar systems are integrated within real-time decision-making systems. This requires a detection method capable of making a quick and accurate prediction while keeping the false-alarm rate as low as possible.
3. **Processing of sequential (stream) data:** As mentioned in Section 2.2, each plot may be correlated with other plots, e.g., when they are part of an identified track. Therefore, in addition to capturing and learning patterns among the features of the same plot record, the model should be able to learn patterns within a sequence of plots.
4. **Explanations of anomalies:** Understanding which parts of data are anomalous can help a system operator perform a correct action. If the method has the ability to identify the packets/features that contribute the most to an anomaly, it can improve a system operator’s decision-making capabilities. The structure of the proposed method enables it to identify an anomaly type detected, determine whether a legitimate message is missing from a sequence of messages, and understand whether a feature of an existing message has been manipulated.

### 5.3. Extracted Features

As described in Section 2.2, each plot record contains a set of features used by a radar system to determine properties with regard to physical objects. In our dataset, each plot record includes 10 categorical features and 18 numerical features, all with a high potential of being exploited by an attacker interested in disrupting a radar system’s normal operation.

**Categorical features.** Five of the categorical features describe a returned signal’s physical properties. The other five are (1) trackType, which specifies the track type, (2) signalQuality, which specifies the quality of a returned signal, (3) objectType, which specifies the type of object detected, (4) alertRaised, which specifies whether an alert has been raised on a system, and (5) objectCategory, which specifies whether an object is considered hostile.

**Numerical features.** Seventeen of the numerical features relate to the correlations between a detected object’s locations. Another numerical feature is timeStamp—the timestamp indicating when a plot has been updated on a system.

Note that due to privacy concerns, a more detailed description of the features in the dataset used in this research cannot be provided. However, Garland et al. [28] proposed a framework for modeling real-time radar systems; this framework can be used for various engineering purposes. For example, the framework can be used to design transferable security techniques, as proposed in this study, to protect radar systems from data manipulation attacks similar to the features used in our work, e.g., track type or a signal’s quality or location. Features containing a physical object’s properties with a high potential of being exploited by attackers can be obtained.

## 6. Low-Level Description of the Proposed Method

In this section, we provide a detailed description of each component of the proposed method: the architectures of the machine learning models, the data preprocessing applied to the raw features, and the procedures proposed for calculating the anomaly thresholds for each module.

### 6.1. Field Manipulation Detection

As illustrated in Figure 3, this module consists of the following two computational components: (1) a plot-level anomaly detector and (2) a track-level anomaly detector. As mentioned earlier, the plot-level anomaly detector generates an anomaly score for each plot independently, while the track-level anomaly detector aggregates the anomaly scores of the plots and generates an alert if a computed value exceeds a certain threshold.

**Figure 4:**
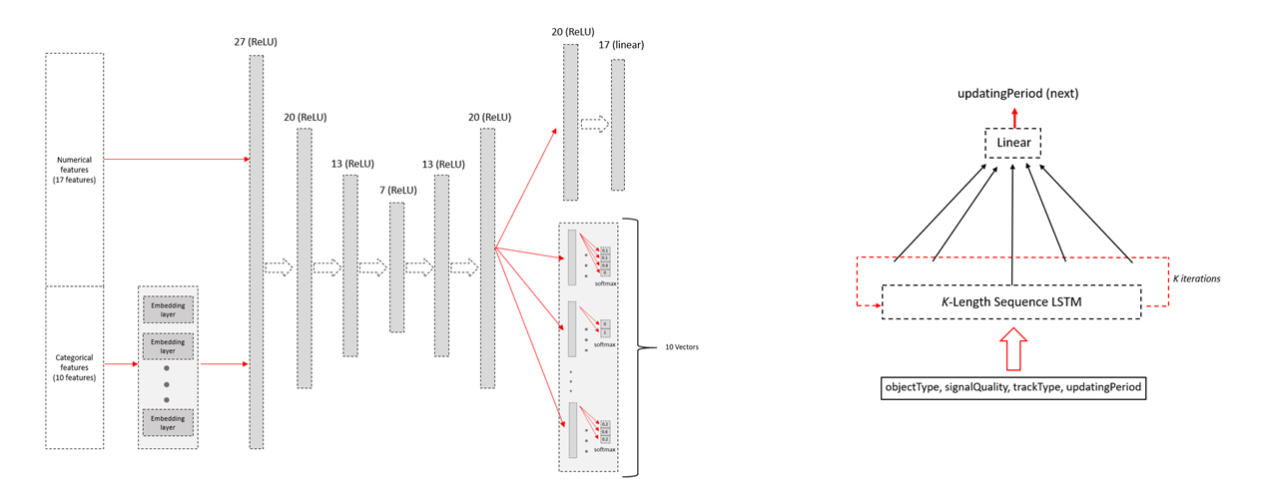
The neural network architecture of the proposed plot-level anomaly detector (**left**) and the neural-network architecture of the proposed sequence-based anomaly detector (**right**).

**Plot-Level Anomaly Detector.** This module focuses on detecting an anomaly within a single plot. The plot features used by this module are described in Section 5.3. In order to detect an anomaly within a given plot, a special variant of an autoencoder is proposed (Figure 4, left side).

Input neurons representing categorical features are attached with an embedding layer. An embedding technique is commonly used to create a concise, numerical representation of categorical features [29]. The embedding representation of the 10 categorical features is concatenated with the 17 numerical features, resulting in a vector of size 27. This vector is then fed to a stacked autoencoder consisting of seven layers with, respectively, 27, 20, 15, 10, 15, 20, and 27 neurons. Finally, the following output layer is attached.

1. For the numerical features, a fully connected layer is attached, followed by a *linear* activation function. This layer contains 17 neurons against 17 numerical features.
2. For each categorical feature with *l* possible values, *l* output neurons are assigned, followed by a softmax function. During inferences, this function provides a probability distribution over all possible values for each categorical feature.

During a training phase, we use data that consist solely of benign tracks. As described in Section 2, each track contains several plots (training examples). We divide the tracks in the dataset so that 80% are used for training and 20% are used for validation. As illustrated in Figure 4, the label for each example is one vector of 17 entries containing the value of each numerical feature, concatenated to 10 vectors representing a one-hot encoding for each of the 10 categorical features. The loss function used is the following combination of the *mean-squared error* (MSE) used for the numerical features (num) and the *sparse categorical cross-entropy* (SCCE) used for the categorical features (cat):

(1) $$loss=MSE\left(x_{i}:x_{i}\in num\right)+\sum_{x_{i}\in cat}SCCE\left(x_{i}\right)$$

Given the training set, the network is trained to minimize the loss function on the validation set using the Adam optimizer. The training is stopped when the loss function reaches its minimum.

**Track-Level Anomaly Detector.** This module consists of a track-score collector and a track-level anomaly detector. The scores generated by the plot-level detection module serve as inputs for this model. The model collects all of the scores for the plots that are part of the same track and outputs them for the track-level anomaly-detector model. All of the plots’ scores for a track serve as the input for this model.

For each track, we define the anomaly score as the average of all of the input scores calculated by the plot-level anomaly detector (they are stored inside the track score collector). In order to determine the anomaly threshold, we used the validation set that was used to train the plot-level anomaly detector. The anomaly threshold was calculated as the maximum value over the tracks’ average scores.

### 6.2. Timing-Based Anomaly Detection

This module focuses on detecting anomalies related to the plots’ arrival times. We observed that timing features by their own were not sufficient to detect such anomalies, and the long term relationships with other features should be learned. Thus, we picked a model that is based on an LSTM, and we used the following subset of plot features (described in Section 5.3): objectType, signalQuality, trackType, and timeStamp. timeStamp was used to extract the time interval between two consecutive plots. The module consists of the following two components: (1) a data preprocessing component and (2) a sequence-based anomaly detector (see Figure 4 (right side)), which is based on an LSTM network.

**Data Preprocessing.** The inputs for this component are the current plot along with the *K* previous plots that correspond to the same track (with a sequence length of K+1).

This component consists of the following three parts:

1. Categorical-feature one-hot encoding: In this part, each feature is converted into a vector that has a value of one in one entry (corresponding to the feature value) and zeros in all of the other places.
2. Plot time interval extraction: In this part, we calculate a feature called updatingPeriod. The value of this feature is the time difference between two consecutive plots in a sequence, e.g., current time–previous time.
3. Feature scaling: In this part, we apply min-max scaling to the data for feature scaling.

**Sequence-Based Anomaly Detector.** This module receives a sequence with a length of K+1 as an input, and by using the first *K* elements of the sequence, the model tries to predict the value of the updatingPeriod, e.g., time interval, feature of the K+1th element, i.e., current plot. To enable this, we designed an LSTM-based regressor. The architecture of the regressor is presented in Figure 4. As can be seen, the *K* length sequence serves as input into an LSTM network with five hidden units. Then, the output of the LSTM component in the final step is fully connected to one neuron. At the end, a linear activation function is applied to this neuron.

During the training phase, we used data consisting solely of benign tracks. For each track, we generated a set of training examples such that each consisted of a set of *K* consecutive plots and was labeled by the updatingPeriod feature of the following K+1th plot. We divided the tracks in the dataset so that 80% were used for training (*T*) and 20% were used for validating (*V*).

The loss function that was used was the MSE. Given *T*, the network was trained to minimize the loss function on *V* using the Adam optimizer. Training was stopped when the loss function reached its minimum.

Once the training was complete, the anomaly threshold thr was set. This anomaly threshold, above which an instance was considered anomalous, was calculated as the sum of the sample mean and standard deviation of MSE over *V*:

(2) $$thr=mean\left(MSE_{V}\right)+std\left(MSE_{V}\right)$$

## 7. Evaluation

For the evaluation, we used benign data collected from four real radar systems that are deployed in different operational setups and are used to identify objects. We refer to each dataset as a *recording session*. The number of messages, i.e., radar data records, in each recording session was as follows: R1: 45,720 (50.7%); R2: 23,017 (25.5%); R3: 11,955 (13.2%); and R4: 9551 (10.6%). In total, there were 90,243 messages.

### 7.1. Simulated Attack Scenarios

Since our dataset did not include any attacks, we had to simulate attacks and inject them into our dataset. We simulated four attack scenarios by manipulating valuable benign data and conducted 15 different experiments for each attack.

**Categorical feature manipulation.** This is an integrity violation attack in which an attacker changes a categorical feature. In the following cases, such manipulations may cause consuming systems or radar operators to incorrectly characterize and handle a detected object.

1. objectType: Changing this feature may cause aerial objects to appear as ground objects and vice versa. This can change the way a radar operator reacts to various objects detected; an attacker can utilize this attack in order to disguise threatening objects, leaving them untreated by radar operators.
2. alertRaised: Changing this feature may cause an alert to be issued for no reason and vice versa. Such an attack can create many false alarms, causing a radar operator to ignore threats.
3. objectCategory: Changing this feature may cause friendly objects to be considered enemy objects and vice versa. This attack can cause radar operators to fire on friendly objects or ignore enemy objects.

Changing the features for an entire track or track segment will be much more beneficial to an attacker than changing the features of random plots. This is because changes in the features of random plots may be ignored by a radar operator and are not likely to influence an action taken by an operator.

Accordingly, to mimic a sophisticated threat against radar, the following process was used to generate a test set given the benign set *B* collected from real radar systems: (1) create $B^{\prime }$ by duplicating *B*; (2) select *f*, the feature that should be manipulated; (3) for each track *T* in $B^{\prime }$, change the value of *f* to a distinct random value from the set of valid values of *f*; (4) label the manipulated plots as anomalies and (5) combine the benign set *B* and the manipulated set $B^{\prime }$ into one test set. This process generates a similar number of benign and malicious plots; therefore, the resulting test set is balanced.

**Plot dropping.** This is an availability violation attack in which an attacker drops one/several plots from a track, so that a detected object will evade a consuming system for some period of time. Dropping several consecutive plots will be much more beneficial to an attacker than dropping nonconsecutive plots or just a single plot. Therefore, for track *T*, we first selected the minimum value *c* (c≤|T|) of plots to be removed from *T*. We picked *c* to be the minimum value that allowed an attacker to cause real harm to the data integrity of a radar system. For the radar system used in our experiments, our *c* was set at 10.

The following process was used to generate the test set given the benign set *B* collected from real radar systems: (1) for each track *T*, select a random plot index *i* and a random integer r∈c,|T|−1; (2) starting at index *i*, drop a minimum number min(|T|−i,r) of consecutive plots; (3) label the plot that follows the dropped plots as an anomaly. This process generated a different number of benign and malicious plots; therefore, the resulting test set is imbalanced.

### 7.2. Evaluation Method

We conducted three types of evaluations; in each case, we repeated the experiment five times (each time a different recording session was selected for testing).

A description of the types of evaluations performed is provided below, along with an example of the training/testing data in a scenario in which the R4 recording session was examined.

- **Cross-session setup:** In this case, each time, we used three recording sessions for training, and the fourth session was used to test the model. For example, for the R4 recording session, training used R1, R2, and R3 and testing used 4.
- **Chronological setup:** In this case, for each recording session, we trained the model on the first 90% of the instances (in chronological order) and tested it on the remaining 10% of the instances. For example, for the R4 recording session, training was 90% of the instances of R4 and testing was the remaining 10% of the instances of R4.
- **Transfer-learning setup:** This case was a combination of the previous two setups in which we used three recording sessions as well as the first 10% of the instances of the fourth session to train the model; the remaining 90% of the instances of the fifth session were used to test the model. For example, for the R4 recording session, training used R1, R2, R3, and the first 10% of the instances of R4; testing used the remaining 90% of the instances of R4.

It should be noted that the cross-session evaluation setup had two significant advantages over the other two: (1) in practice, it is easier for radar engineers to deploy a model that has already been trained rather than training a model using new system data, and (2) training a model on new system data can expose the model to cyber risks, e.g., adversarial poisoning [30].

### 7.3. Evaluation Metrics

The task of identifying anomalies in the radar data stream was a binary classification task in which benign samples were labeled as zero (negative) and malicious data was labeled as one (positive). We used the following common metrics for the evaluation: true positive rate (TPR)/recall, false positive rate (FPR), receiver-operating characteristic (ROC) curve, area under the ROC curve (AUC), precision, precision-recall (PRC) curve, and average precision (AP).

### 7.4. Results

In this section, we present the evaluation results for each attack scenario (presented in Section 7.1) and evaluation method (presented in Section 7.2). First, we present a graph of the evaluation results as a function of different thresholds (using ROC and PRC graphs). Then, we summarize the evaluation results for predefined anomaly thresholds. The method for calculating the anomaly thresholds is described in Section 6.

**Figure 5:**
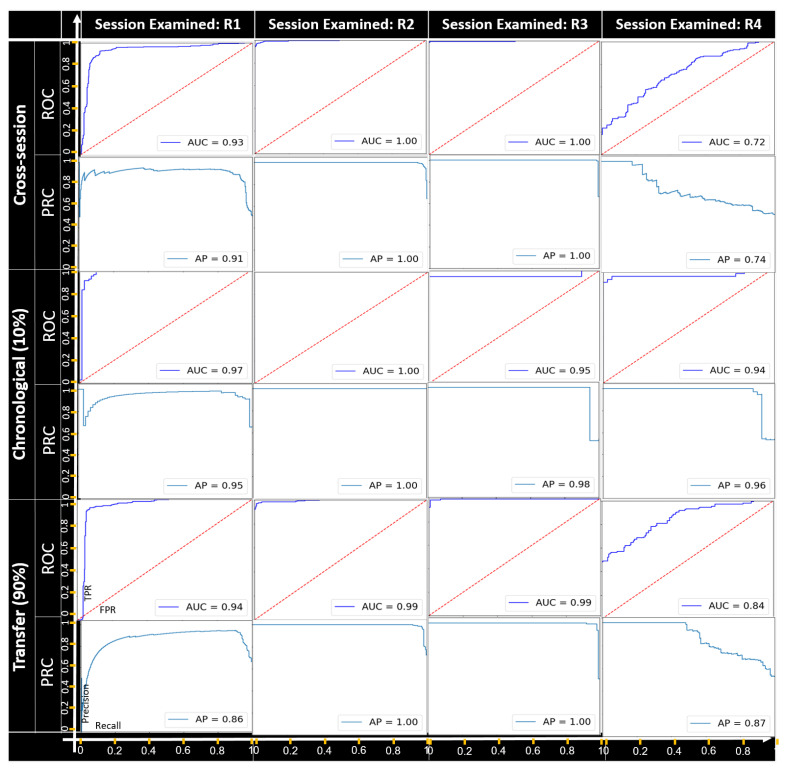
The ROC and PRC curves for the objectType feature-manipulation attack for each recording session examined.

**Figure 6:**
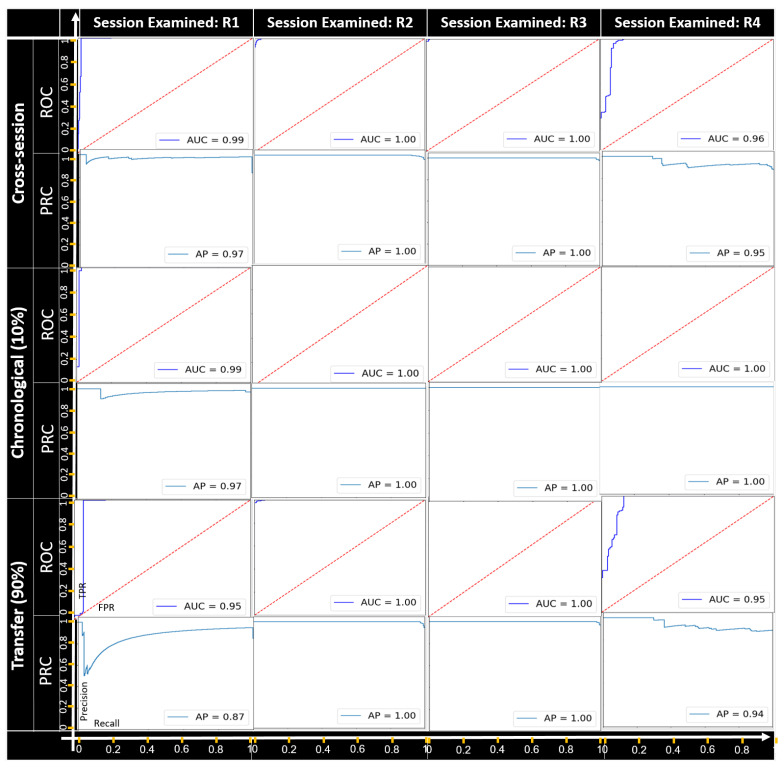
The ROC and PRC curves for the objectCategory feature-manipulation attack for each recording session examined.

**Figure 7:**
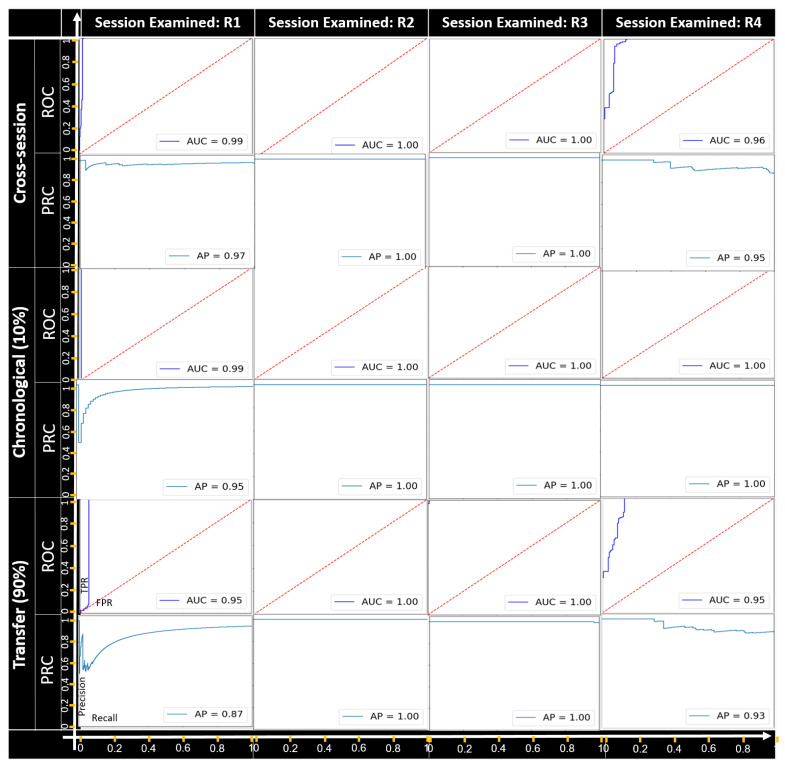
The ROC and PRC curves for the alertRaised feature-manipulation attack for each recording session examined.

**Categorical feature manipulation.**Figure 5, Figure 6 and Figure 7 present the detection results of the field manipulation-detection module when manipulating the objectType, objectCategory, and alertRaised features, respectively; the ROC curve shows the true positive rate (TPR) and false positive rate (FPR) for every possible anomaly detection threshold, and the AUC provides an overall performance. The PRC curve shows the precision and recall for every possible anomaly detection threshold, and the AP provides an overall performance. As can be seen, in all cases except for recording session R4 of the objectType feature-manipulation attack, all the setups had a great performance in terms of the AUC and AP for all the categorical feature-manipulation attacks.

For recording session R4 of the objectType feature, the best performance was obtained with the chronological setup; this indicates that the ability to detect manipulation attacks becomes weaker when using a pretrained model (without retraining) for cases in which the objectType feature has been manipulated by an attacker. As can be seen, the best detection rates were observed for the manipulation attack on both the objectCategory and alertRaised features.

**Figure 8:**
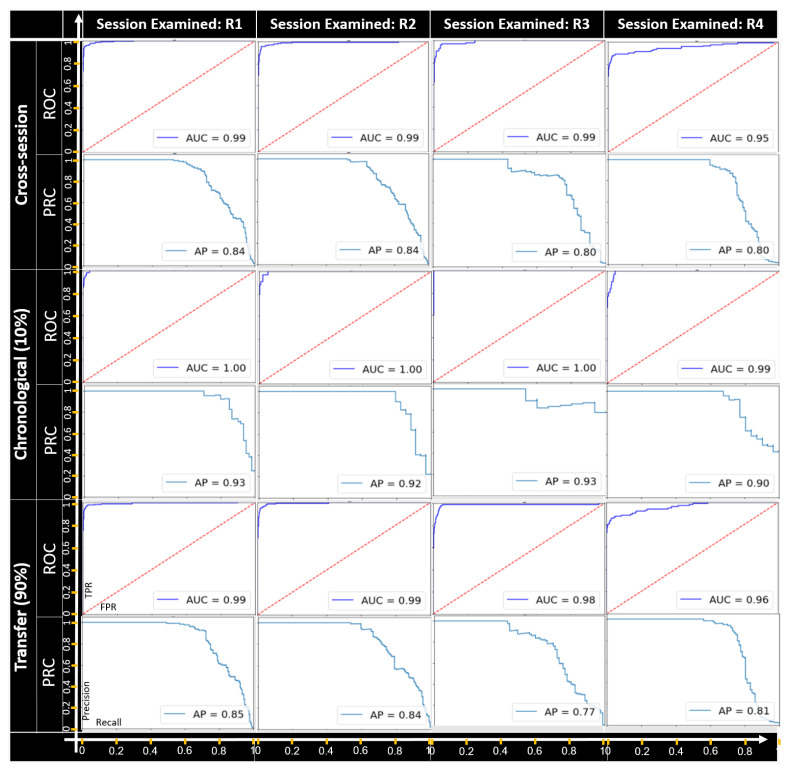
The ROC and PRC curves for the Plot-dropping attack for each recording session examined.

**Plot dropping.**Table 1 presents the distribution of the benign malicious samples in the plot-dropping attack. It should be noted that the data were imbalanced since for each track, we only executed the attack once. The performance of the timing-based anomaly-detection module on the generated test set is presented in Figure 8. During our experiments, the *K* parameter representing the length of the plot sequence used by the sequence-based anomaly-detector module was optimized to the value of five. As can be seen, in all scenarios, the model achieved good results; the AUC provided an overall performance for every possible detection threshold. Thus, as can be seen, our proposed method achieved very good results for the cross-session setup; this indicates the ability of the proposed method to generalize to different setups.

**Performance for predefined anomaly thresholds.**Table 2 presents the performance for each attack scenario and each setup in terms of the true-positive rate (TPR) and false-positive rate (FPR) for a threshold that was computed on a validation set and then applied on a test set. As can be seen, our proposed method can detect 90% of the plot-dropping attacks with a false-positive rate of 2% and can detect from 76% to 96% of feature-manipulation attacks with a false-positive rate of 2%. In typical scenarios, 400 plots are generated and transferred by a radar system every minute. A false-alarm rate of 2% means that eight plots should be tracked by a system operator/automatic system every minute.

In addition, in most cases, our proposed method achieved very good results for the cross-session setup. This is an important point, since it means that a pretrained model can be migrated to new radar systems without retraining.

### 7.5. Performance Analysis

In order to understand whether the proposed method is practical and can be applied to the real-time tracking of anomalies in radar-system data streams, we measured the average time it takes to process a single plot. This experiment was conducted on a standard machine with an Intel core i7-7600U 2.8 Ghz CPU and 32 GB of RAM. The machine’s operating system was Windows 10.

The results showed that our proposed method can analyze 20,000 plots per second. Given that in typical scenarios, a radar system generates and transfers 400 plots per minute, we concluded that our method is practical and can be used for the real-time tracking of anomalies in radar-system data streams.

## 8. Summary and Conclusions

In this paper, we propose a novel deep-learning-based method for detecting data manipulation attacks on radar systems. We considered attack scenarios in which an attacker manipulates the data sent by a radar system to peripheral consuming systems. To evaluate our method, we used data collected from four real radar systems. This dataset consisted of plot records that were sent from a radar system to a peripheral consuming system.

To evaluate our method, we generated attacks considered beneficial to an attacker, ranging from integrity-violation attacks to availability-violation attacks. The results of our evaluation showed that the proposed method can learn the normal behavior of the data and distinguish between normal and manipulated data.

In our design process, we took into account the benefits of identifying an anomaly type detected, determining whether a legitimate plot is missing from a sequence of plots, and understanding whether an existing plot’s feature has been manipulated; these properties could help radar operators determine the correct actions to take when attacks occur.

The transferability and practicality of the proposed method was demonstrated as follows: (1) our method obtained very good results when training on data collected from three radar systems and testing on data collected from another radar system, and (2) a typical Intel controller can analyze plots at a frequency of 20,000/s, while radar systems generate and transfer plots at a frequency of 7/s.

In future research, we plan to extend the proposed method with an explainability mechanism that may be a good basis for real-time remediation and thus for potentially preventing the malicious effects of attacks.

## Figures and Tables

**Table 1:** Numbers of benign (b) and malicious (m) samples in the plot dropping attack.

	Recording Session Examined							
	R1		R2		R3		R4	
	b	m	b	m	b	m	b	m
Cross-session	46,600	213	23,068	177	12,139	97	9484	119
Chron. (10%)	4606	60	2323	34	1206	15	842	31
Transfer (90%)	42,709	195	21,126	170	10,808	86	8451	110

**Table 2:** Evaluation results obtained for predefined anomaly thresholds.

Setup	Avg. AUC	Avg. PR	Avg. TPR	Avg. FPR
**Feature manipulation of objectType**				
Cross-session	0.922	0.930	0.759	0.028
Chronological (10%)	0.972	0.712	0.894	0.016
Transfer (90%)	0.943	0.931	0.710	0.090
**Feature manipulation of objectCategory**				
Cross directories	0.986	0.973	0.906	0.028
Chronological (10%)	0.997	0.994	0.770	0.016
Transfer (90%)	0.975	0.952	0.769	0.090
**Feature manipulation of alertRaised**				
Cross-session	0.987	0.975	0.963	0.028
Chronological (10%)	0.997	0.988	0.776	0.016
Transfer (90%)	0.974	0.950	0.750	0.090
**Plot dropping**				
Cross-session	0.975	0.820	0.900	0.023
Chronological (10%)	0.987	0.916	0.937	0.026
Transfer (90%)	0.983	0.830	0.909	0.023

## Data Availability

The data are not publicly available due to security concerns.
